# Dose–response efficacy and safety of PA21 in Japanese hemodialysis patients with hyperphosphatemia: a randomized, placebo-controlled, double-blind, Phase II study

**DOI:** 10.1007/s10157-016-1299-z

**Published:** 2016-07-07

**Authors:** Fumihiko Koiwa, Akira Terao

**Affiliations:** 1 Division of Nephrology, Department of Medicine, Showa University Fujigaoka Hospital, 1-30 Fujigaoka, Aoba-ku, Yokohama, 227-8501 Japan; 2 Biostatistics, Faculty of Pharmaceutical Sciences, Josai University, Sakado, Japan

**Keywords:** Hemodialysis, Hyperphosphatemia, Japanese, PA21 compound, Phosphate binder, Sucroferric oxyhydroxide

## Abstract

**Background:**

Hyperphosphatemia is common in chronic kidney disease (CKD) and associated with mortality and morbidity. We aimed to evaluate the dose-dependent efficacy and safety of PA21 (sucroferric oxyhydroxide), an iron-based phosphate binder, in Japanese hemodialysis patients with hyperphosphatemia.

**Methods:**

In this double-blind, multicenter, Phase II study, 183 patients were randomized to placebo or PA21 at doses of 250, 500, 750, or 1000 mg (based on iron content) three times/day for 6 weeks. The primary efficacy endpoint was the mean change in serum phosphorus levels from baseline to end of treatment in each group. Adverse reactions were evaluated.

**Results:**

The change in serum phosphorus level was significantly greater in each PA21 group than in the placebo group (analysis of covariance: *P* < 0.001 for all groups). A dose-dependent change in serum phosphorus levels was observed in the PA21 groups. A notable decrease in mean serum phosphorus levels to the target level of ≤6 mg/dL was shown starting at Week 1 in all PA21 groups. The cumulative achievement rates for target serum phosphorus level at the end of treatment were generally >80 % in all PA21 groups. The major adverse reaction reported was diarrhea; however, most cases were mild.

**Conclusions:**

PA21 was an effective and safe treatment that decreased serum phosphorus levels starting at 1 week of treatment when administered as one 250-mg tablet three times/day. PA21 demonstrated a dose-dependent phosphorus lowering effect up to 3000 mg/day. PA21 may be a new treatment alternative with relatively low pill burden for Japanese hemodialysis patients with hyperphosphatemia.

## Introduction

Dietary phosphate is absorbed from the gastrointestinal tract and excreted mainly through the kidneys. Eventually, hyperphosphatemia occurs in end-stage renal disease, as a result of phosphorus accumulation in the body. Additionally, bone mineral (e.g., calcium and phosphorus) metabolism abnormalities associated with chronic kidney disease (CKD) are designated as CKD-mineral and bone disorder, which is a systemic disease that influences the prognosis by affecting not only bones but also blood vessels and accelerating medial arterial calcification and arteriosclerosis [1, 2]. It is known that hyperphosphatemia causes and/or worsens ectopic calcification and secondary hyperparathyroidism. Furthermore, increased serum phosphorus levels have been reported to be an independent prognostic factor for mortality [3, 4]. Therefore, management of serum phosphorus in chronic hemodialysis patients is recommended in CKD management guidelines in many countries including Japan [1, 5, 6]. Although dietary therapy is the basic treatment for patients with CKD, and a certain amount of phosphorus can be eliminated by hemodialysis, excessive protein restriction causes malnutrition and may increase the risk of death [7], and conventional hemodialysis cannot completely eliminate phosphorus excess. Thus, in addition to dietary therapy and hemodialysis, oral phosphate binders are needed to inhibit the absorption of dietary phosphate and prevent vascular calcifications in CKD patients [8, 9].

The use of sevelamer hydrochloride, lanthanum carbonate, and bixalomer was approved in the 2000s in Japan, in addition to conventional calcium carbonate, to prevent the excessive phosphorus absorption in chronic hemodialysis patients. Ferric citrate was introduced in 2014. Although calcium carbonate is widely used for its effectiveness, some concerns have been raised regarding hypercalcemia and vascular calcification, which are caused by the uptake of calcium contained in the drug [10, 11]. Sevelamer hydrochloride and bixalomer, both of which are non-absorbed polymers containing no calcium, do not raise concerns related to calcium absorption. However, in Japan, sevelamer hydrochloride is only available in the form of 250-mg tablets, and patients are required to take at least three or four tablets/dose to control their serum phosphorus levels in clinical practice. Furthermore, a high incidence of gastrointestinal adverse reactions has been reported in patients treated with these drugs, such as abdominal distension and constipation [12]. Regarding lanthanum carbonate, its effect can be obtained with a smaller number of tablets, but it causes a high incidence (approximately 10 %) of vomiting and nausea [13]. Some concerns have been raised about the absorption and its effects on the long-term safety of lanthanum, which is a rare metal. Moreover, regarding the use of ferric citrate, there is concern about the elevation of serum ferritin levels [14].

PA21, a mixture of polynuclear iron (III) oxyhydroxide, sucrose, and starches, is a novel non-calcium-based phosphate binder [15]. An in vitro study showed that the phosphate binding capacity of PA21 was over a physiologically relevant pH range in the gastrointestinal tract [16]. In a Phase III study conducted in multiple sites across Europe, the United States, Russia, Ukraine, and South Africa, the phosphorus-lowering effect of PA21 was shown by demonstrating its non-inferiority to sevelamer carbonate and superiority to an ineffective control [17]. An extension of the Phase III study confirmed the long-term efficacy of PA21 which was maintained over 1 year [18]. Additionally, PA21 has been launched in Europe and the United States, among other regions and countries. The present study aimed to evaluate the dose-dependent efficacy and safety of PA21 in Japanese hemodialysis patients with hyperphosphatemia.

## Methods

### Subjects

The inclusion criteria were as follows: men and women ≥20 years of age at the time consent was obtained who were considered able to discontinue their current hyperphosphatemia therapy for the 3-week wash-out period; subjects who, before the wash-out period, were undergoing maintenance hemodialysis three times weekly for ≥12 weeks and planned to continue hemodialysis during the study treatment period, did not undergo changes in the dose of phosphate binder agent, vitamin D receptor activator or vitamin D receptor modulator, calcium sensing receptor agonist of calcimimetics, or anti-osteoporotic agents for ≥4 weeks, or did not present changes in the dialysate calcium concentration for ≥4 weeks; and those with serum phosphorus concentration >6.0 and ≤10 mg/dL at the first dialysis session in the wash-out period (Week-1).

The main exclusion criteria were as follows: subjects who, before the first dialysis session of the wash-out period, had a corrected serum calcium concentration ≤7.5 or >11.0 mg/dL, an intact parathyroid hormone (PTH) concentration of >800 pg/mL, or a concentration >500 pg/mL if determined to have poor control; history of hemochromatosis, any other iron overload disorder, serum ferritin >800 ng/mL, or transferrin saturation >50 %; subjects planning to undergo parathyroidectomy or percutaneous ethanol injection therapy (PEIT) during the study period, or who underwent parathyroidectomy or PEIT ≤24 weeks before their wash-out period; clinically significant gastrointestinal disorder or history of a clinically significant digestive tract procedure according to the investigator’s diagnosis; clinically significant hepatic disorder (e.g., alanine transaminase or aspartate transaminase ≥100 U/L or total bilirubin ≥3.0 mg/dL); and history of brain/cardiovascular disorder (e.g., myocardial infarct, unstable angina, cerebral infarct, cerebral hemorrhage). Other phosphate binders, agents with a phosphorus adsorption effect, agents that affect serum phosphorus levels, and intravenous and oral iron therapies were not permitted. Patients were withdrawn if their serum phosphorus levels exceeded 10 mg/dL or decreased below 3.0 mg/dL during two consecutive evaluations, or if their corrected serum calcium levels decreased below 7.5 mg/dL.

The present study was approved by the institutional review board of each participating center (approval number: PA1201) and performed in accordance with the Helsinki Declaration of 1964, as revised in 2000. Written informed consent was obtained from all individual participants included in the study.

### Study design and treatment

This Phase II, randomized, placebo-controlled, double-blind comparative study included hemodialysis patients from 14 centers in Japan. The present study had a 3-week wash-out period and a 6-week treatment period in which placebo or PA21 doses of 250, 500, 750, or 1000 mg were orally administered three times/day prior to meals. The subjects were randomized to the following five groups: 750-, 1500-, 2250-, 3000-mg/day, and placebo group. Each PA21 tablet contained 250 mg of iron. The placebo tablet did not contain active moiety. Randomization of subjects was carried out by interactive web response system with subjects randomized in a 1:1:1:1:1 ratio to one of the four PA21 groups or the placebo group. The trial was registered on ClinicalTrials.gov (NCT01521494).

### Efficacy endpoints

The primary efficacy endpoint was the mean change in serum phosphorus levels from baseline to end of treatment in each treatment group. Additional evaluations of the primary endpoint included serum phosphorus level at each time-point and cumulative achievement rates for target serum phosphorus level defined as the proportion of patients with serum phosphorus level of ≤6 mg/dL at each time-point according to the upper limit of the target range of the Japanese Society for Dialysis Therapy (JSDT) guidelines [6]. The secondary efficacy endpoints were corrected serum calcium levels and serum intact-PTH levels.

### Safety and tolerability

Safety outcomes included adverse events (AEs), adverse drug reactions (ADRs), laboratory tests (including ferritin, transferrin saturation, and hemoglobin level), physical examination (e.g., vital signs), and 12-lead electrocardiography (ECG) findings.

### Statistical analysis

The planned sample size was 150 subjects (30 per group) to have a 90 % power to detect a 1.6 mg/dL difference in serum phosphorus level between the PA21 and placebo groups. This calculation assumed a standard deviation of 1.8 mg/dL using an alpha level of 0.05. The primary efficacy endpoint and serum phosphorus concentration at each time-point were summarized by dose group using descriptive statistics. The primary endpoint was compared between the treatment and placebo groups using analysis of covariance (ANCOVA) with baseline serum phosphorus levels as covariates. For the primary efficacy endpoint, a closed testing procedure was used to allow multiple statistical comparisons between each PA21 group and the placebo group while maintaining the Type I error rate at 0.05. Cumulative achievement rates for target serum phosphorus levels were calculated per group at each evaluation time. Comparisons between the placebo group and each PA21 group were made using Fisher’s exact test. Descriptive statistics were used for each secondary endpoint measure by group at each evaluation time. The number and incidences of AEs and ADRs were calculated in each group. The AEs and ADRs did not include discoloration events caused by the iron contained in PA21 and were classified according to (MedDRA) primary System Organ Class and Preferred Terms. Statistical significance was set at *P* < 0.05. All statistical analyses were performed using SAS 9.3 Foundation for Microsoft Windows (SAS Institute Inc., Cary, NC, USA).

## Results

### Subjects

**Fig. 1:**
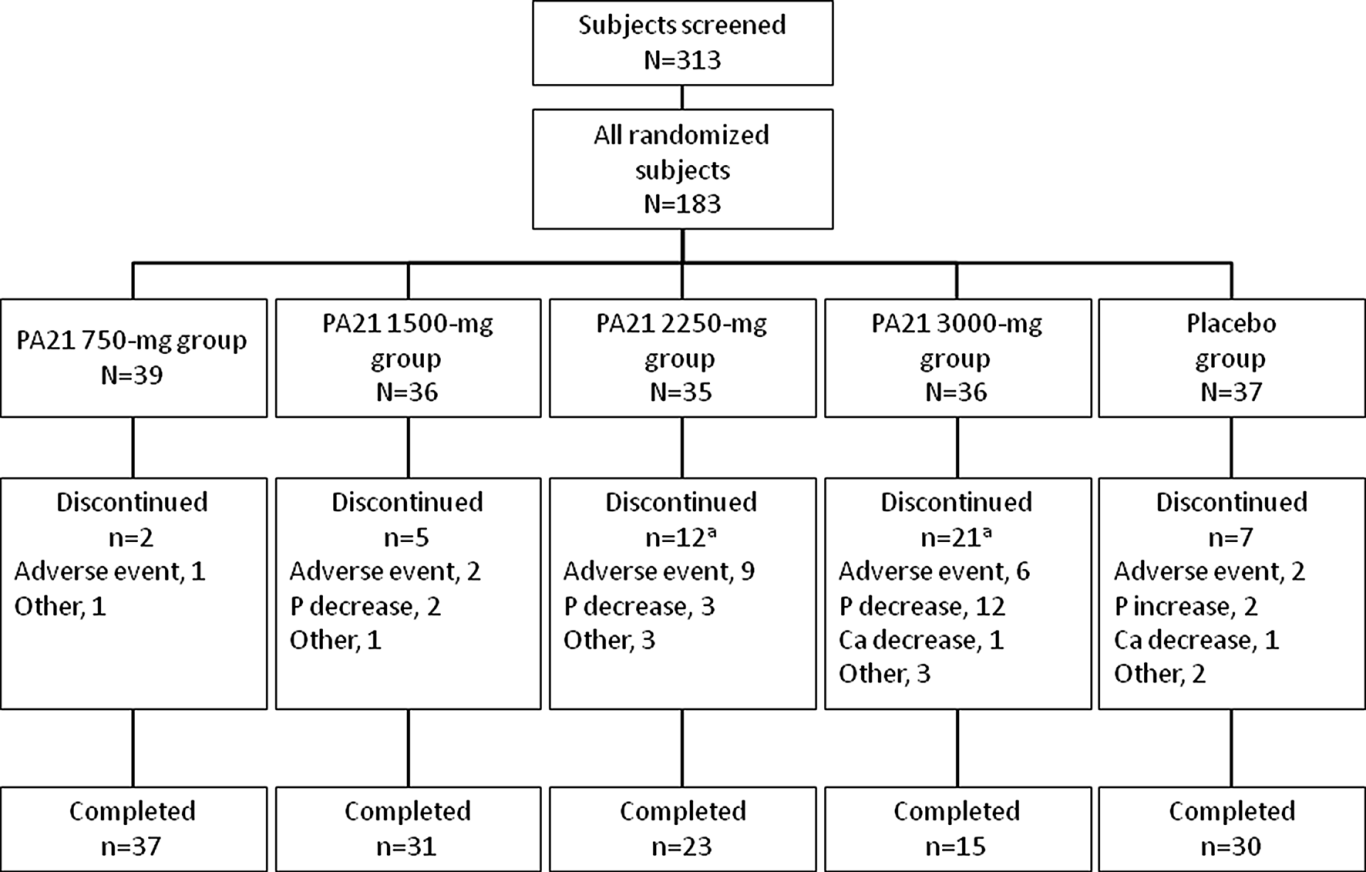
Disposition of patients. Some subjects had more than one reason for discontinuation. *Abbreviations* *Ca* calcium, *P* phosphorus

A total of 313 patients were screened in this study, and eligible 183 subjects were randomized to the placebo and PA21 groups. The disposition of patients is shown in Fig. 1. A total of 178 and 183 subjects were included in the full analysis set and safety analysis set, respectively. Of the 183 patients randomized, the proportion of subjects who discontinued treatment was 18.9 % (7/37) in the placebo group and 5.1 % (2/39), 13.9 % (5/36), 34.3 % (12/35), and 58.3 % (21/36) in the 750-, 1500,- 2250-, and 3000-mg PA21 groups, respectively. The reasons for discontinuation in each treatment group are shown in Fig. 1. Seventeen patients discontinued treatment because their serum phosphorus levels decreased to below the defined criterion for discontinuation. Among them, 15 patients received higher doses of PA21 (three patients in the 2250-mg group and 12 patients in the 3000-mg group). Eleven patients discontinued treatment because of diarrhea. Of these, nine patients received higher doses of PA21 (five patients in the 2250-mg group and four patients in the 3000-mg group). In all the patients, the diarrhea was resolved with discontinuation of the investigational product. In total, 136 patients completed the study (106 in the PA21 groups and 30 in the placebo group).

The background characteristics of patients are shown in Table 1. The five groups were generally comparable. Among the 178 subjects analyzed (full analysis set), the mean ± standard deviation age was 61.4 ± 10.9 years and 115 were men (64.6 %).

**Table 1:** Patient background characteristics (full analysis set)

Characteristic	Overall (*N* = 178)	PA21 750-mg group (*N* = 39)	PA21 1500-mg group (*N* = 35)	PA21 2250-mg group (*N* = 33)	PA21 3000-mg group (*N* = 34)	Placebo group (*N* = 37)
Sex						
Men	115 (64.6)	27 (69.2)	23 (65.7)	23 (69.7)	19 (55.9)	23 (62.2)
Women	63 (35.4)	12 (30.8)	12 (34.3)	10 (30.3)	15 (44.1)	14 (37.8)
Age (years)	61.4 ± 10.9	59.4 ± 10.4	63.8 ± 12.0	61.9 ± 10.5	61.4 ± 11.2	60.8 ± 10.2
Primary disease (*n*)						
Diabetic nephropathy	52	14	9	5	10	14
Chronic glomerulonephritis	56	12	8	11	14	11
Nephrosclerosis	23	7	6	6	1	3
Polycystic kidney disease	10	2	2	2	1	3
Chronic pyelonephritis	1	0	0	1	0	0
Others	11	0	2	3	3	3
Unknown	26	4	8	6	5	3
Mode of dialysis						
Hemodialysis	176 (98.9)	38 (97.4)	35 (100.0)	32 (97.0)	34 (100.0)	37 (100.0)
Hemodiafiltration	2 (1.1)	1 (2.6)	0 (0)	1 (3.0)	0 (0)	0 (0)
Dialysis vintage (months)	83.7 ± 63.5	77.6 ± 67.5	85.1 ± 60.7	95.8 ± 81.9	91.5 ± 58.6	71.0 ± 45.0
Serum phosphorus (mg/dL)	7.46 ± 1.22	7.36 ± 1.18	7.69 ± 1.32	7.42 ± 0.87	7.57 ± 1.33	7.26 ± 1.35
Corrected serum calcium (mg/dL)	8.60 ± 0.56	8.49 ± 0.46	8.65 ± 0.52	8.59 ± 0.66	8.69 ± 0.52	8.58 ± 0.61
Intact parathyroid hormone (pg/mL)	285.3 ± 151.1	277.9 ± 148.9	262.1 ± 139.7	345.0 ± 176.1	262.8 ± 143.3	282.4 ± 140.5

Data are presented as *n* (%) or mean ± SD, unless otherwise stated

Fisher’s exact text was used to determine differences in sex. One-way analysis of variance was used to determine differences in age and serum phosphorus, calcium, and intact parathyroid hormone levels between the groups

### Primary efficacy outcome and additional evaluations

**Fig. 2:**
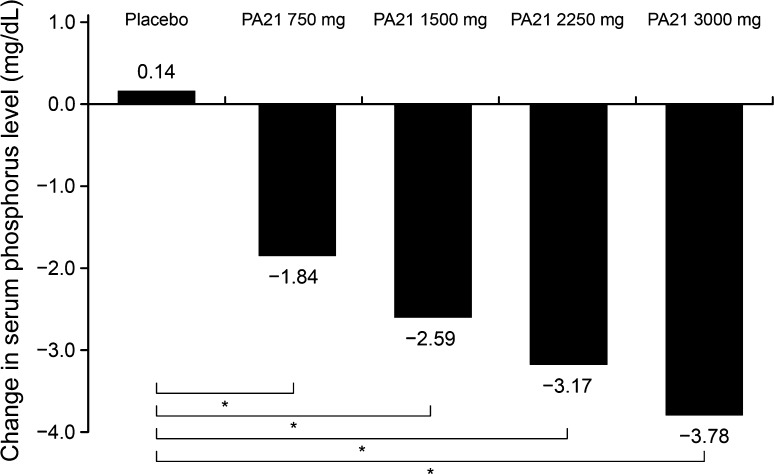
Change from baseline to end of treatment in serum phosphorus levels (full analysis set). The changes in serum phosphorus levels from baseline to end of treatment are adjusted for serum phosphorus levels at baseline. ANCOVA: * *P* < 0.001 for all PA21 groups vs placebo

The mean changes in serum phosphorus levels from baseline to end of treatment (adjusted for serum phosphorus levels at baseline) are shown in Fig. 2. The change in serum phosphorus level in each PA21 group was significantly greater than that in the placebo group (ANCOVA with serum phosphorus levels at baseline as covariates: *P* < 0.001 for all groups). A dose-dependent change in serum phosphorus levels in the PA21 groups was observed. Good compliance to treatment with the study drug was seen throughout the treatment; moreover, there were no changes in the protein catabolic rate and Kt/V (*K*, clearance; *t*, dialysis time; *V*, urea distribution volume) from baseline to end of treatment (Table 2).

**Table 2:** Serum phosphorus, corrected calcium, intact-parathyroid hormone (PTH) levels, protein catabolic rate and Kt/V at each time-point

	PA21 750-mg group	PA21 1500-mg group	PA21 2250-mg group	PA21 3000-mg group	Placebo group
Serum phosphorus level, mg/dL, mean ± SD (*N*)					
Week-3	5.71 ± 1.22 (39)	5.75 ± 1.08 (35)	5.65 ± 0.93 (33)	5.33 ± 1.03 (34)	5.58 ± 1.08 (37)
Week 0	7.36 ± 1.18 (39)	7.69 ± 1.32 (35)	7.42 ± 0.87 (33)	7.57 ± 1.33 (34)	7.26 ± 1.35 (37)
End of treatment	5.57 ± 1.58 (39)	4.99 ± 1.19 (35)	4.27 ± 1.14 (33)	3.74 ± 1.17 (34)	7.50 ± 1.72 (37)
Change from Week 0 to end of treatment	−1.79 ± 1.40 (39)	−2.70 ± 1.30 (35)	−3.15 ± 1.36 (33)	−3.84 ± 1.59 (34)	0.24 ± 1.22 (37)
Corrected serum calcium level, mg/dL, mean ± SD (*N*)					
Week-3	8.83 ± 0.50 (39)	8.99 ± 0.59 (35)	8.97 ± 0.51 (33)	9.06 ± 0.67 (34)	8.85 ± 0.64 (37)
Week 0	8.49 ± 0.46 (39)	8.65 ± 0.52 (35)	8.59 ± 0.66 (33)	8.69 ± 0.52 (34)	8.58 ± 0.61 (37)
End of treatment	8.69 ± 0.49 (39)	8.81 ± 0.49 (35)	8.97 ± 0.58 (33)	9.07 ± 0.65 (34)	8.49 ± 0.63 (37)
Change from Week 0 to end of treatment	0.20 ± 0.34 (39)	0.16 ± 0.33 (35)	0.38 ± 0.44 (33)	0.38 ± 0.39 (34)	−0.09 ± 0.31 (37)
Serum intact-PTH level, pg/mL, mean ± SD (*N*)					
Week-3	198.5 ± 147.9 (39)	187.7 ± 134.0 (35)	249.8 ± 186.7 (33)	169.3 ± 95.4 (34)	195.4 ± 97.5 (37)
Week 0	277.9 ± 148.9 (39)	262.1 ± 139.7 (35)	345.0 ± 176.1 (33)	262.8 ± 143.3 (34)	282.4 ± 140.5 (37)
End of treatment	242.7 ± 159.0 (39)	221.2 ± 141.3 (34)	261.7 ± 168.1 (31)	173.4 ± 96.6 (31)	303.9 ± 170.1 (37)
Change from Week 0 to end of treatment	−35.2 ± 89.8 (39)	−45.6 ± 79.2 (34)	−97.0 ± 92.7 (31)	−86.5 ± 104.0 (31)	21.5 ± 82.5 (37)
Protein catabolic rate (G/kg/day), mean ± SD					
Week 0	0.939 ± 0.121 (39)	1.000 ± 0.136 (35)	0.973 ± 0.159 (33)	0.936 ± 0.157 (34)	0.951 ± 0.166 (37)
End of treatment	0.926 ± 0.153 (38)	0.954 ± 0.101 (33)	0.892 ± 0.142 (33)	0.831 ± 0.182 (33)	0.931 ± 0.159 (37)
Kt/V, mean ± SD					
Week 0	1.419 ± 0.292 (39)	1.471 ± 0.298 (35)	1.478 ± 0.300 (33)	1.499 ± 0.292 (34)	1.430 ± 0.247 (37)
End of treatment	1.438 ± 0.323 (38)	1.452 ± 0.285 (33)	1.464 ± 0.277 (33)	1.484 ± 0.285 (33)	1.429 ± 0.254 (37)

**Fig. 3:**
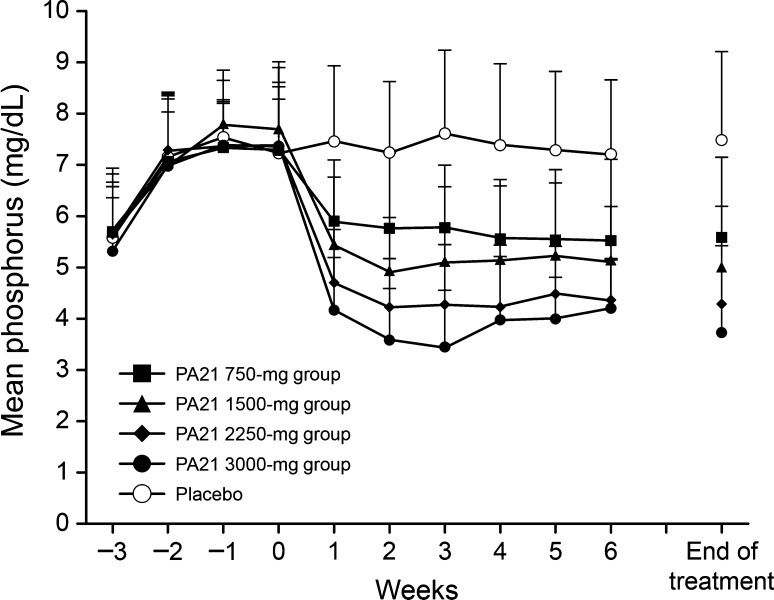
Time course of mean serum phosphorus levels (full analysis set). The mean serum phosphorus levels were significantly higher in all the PA21 groups in all the time-points after administering the PA21 dose (all *P* < 0.001, one-sample *t* test)

The time course of mean serum phosphorus levels in the full analysis set is shown in Fig. 3. A notable decrease in mean serum phosphorus levels to the target serum phosphorus level of ≤6 mg/dL was shown as early as Week 1 in all PA21 groups. This decreasing tendency was maintained until Week 6 (end of treatment).

**Fig. 4:**
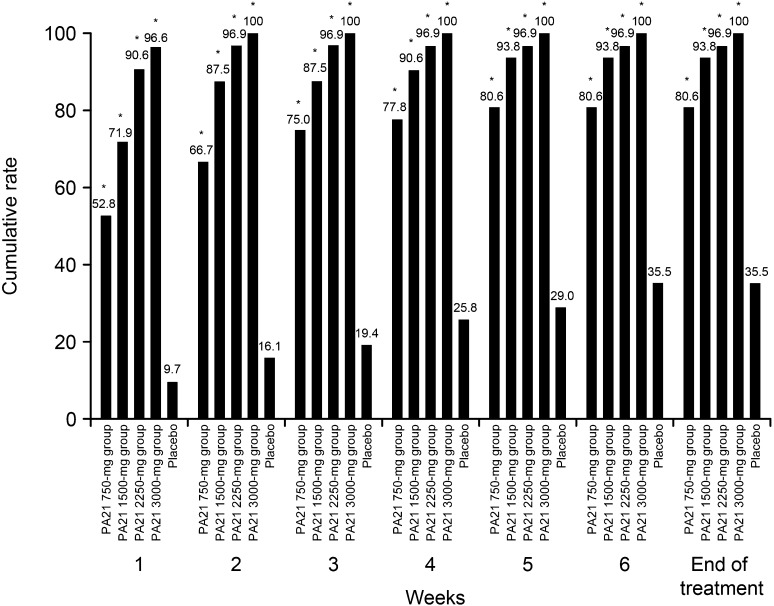
Cumulative achievement rates of target serum phosphorus levels (≤6.0 mg/dL) (full analysis set). The numbers of patients analyzed in the 750-, 1500-, 2250-, 3000-mg, and placebo group were 36, 32, 32, 29, and 31, respectively. The cumulative achievement rates were significantly higher in all the PA21 groups than in the placebo group (all * *P* < 0.001, Fisher’s exact test)

The cumulative achievement rates for target serum phosphorus level (≤6 mg/dL) at each time-point are shown in Fig. 4. The cumulative achievement rates were significantly higher in all the PA21 groups than in the placebo group (all *P* < 0.001, Fisher’s exact test).

### Secondary efficacy outcomes

No significant changes were observed in the corrected serum calcium levels. Although the intact-PTH levels increased from Week-3 to Week 0 (baseline) because of the wash-out of the phosphate binder, the levels at the end of treatment decreased to those measured at the wash-out period (Table 2).

### Safety and tolerability

The incidence of AEs was 54.1 % (20/37) in the placebo group and 61.5 % (24/39), 44.4 % (16/36), 77.1 % (27/35), and 72.2 % (26/36) in the 750-, 1500-, 2250-, and 3000-mg PA21 groups, respectively. The incidence of ADRs was 10.8 % (4/37) in the placebo group and 23.1 % (9/39), 13.9 % (5/36), 40.0 % (14/35), and 44.4 (16/36) in the 750-, 1500-, 2250-, and 3000-mg PA21 groups, respectively. The most frequently reported AEs and ADRs (incidence ≥5 % in any PA21 group) are shown in Table 3. Diarrhea was reported more frequently in the PA21 groups. However, most cases were mild, and more than half of the cases occurred within 2 days after dose initiation. The time to onset of diarrhea is shown in Table 4.

**Table 3:** Adverse events and adverse drug reactions that occurred at an incidence of ≥5 % in the PA21 groups

	PA21 750-mg group (*N* = 39)	PA21 1500-mg group (*N* = 36)	PA21 2250-mg group (*N* = 35)	PA21 3000-mg group (*N* = 36)	Placebo group (*N* = 37)
Adverse events					
Diarrhea	6 (15.4)	6 (16.7)	13 (37.1)	15 (41.7)	7 (18.9)
Contusion	0 (0.0)	0 (0.0)	0 (0.0)	4 (11.1)	0 (0.0)
Nasopharyngitis	5 (12.8)	5 (13.9)	3 (8.6)	3 (8.3)	4 (10.8)
Constipation	0 (0.0)	1 (2.8)	2 (5.7)	2 (5.6)	1 (2.7)
Abdominal pain	0 (0.0)	0 (0.0)	0 (0.0)	2 (5.6)	0 (0.0)
Pain in extremity	0 (0.0)	1 (2.8)	2 (5.7)	0 (0.0)	0 (0.0)
Hemorrhoids	0 (0.0)	0 (0.0)	2 (5.7)	0 (0.0)	0 (0.0)
Insomnia	2 (5.1)	0 (0.0)	0 (0.0)	0 (0.0)	0 (0.0)
Upper respiratory tract inflammation	2 (5.1)	0 (0.0)	0 (0.0)	0 (0.0)	0 (0.0)
Adverse drug reactions					
Diarrhea	4 (10.3)	4 (11.1)	12 (34.3)	12 (33.3)	3 (8.1)
Constipation	0 (0.0)	1 (2.8)	2 (5.7)	1 (2.8)	0 (0.0)

Data are presented as *n* (%)

**Table 4:** Time to onset of diarrhea

Time to onset	PA21 groups	Placebo group
1–7 days	31	4
1st day	6	0
2nd day	10	1
3rd day	8	1
4th day	2	0
5th day	5	1
6th day	0	1
7th day	0	0
8–14 days	3	0
15–21 days	2	2
22–28 days	2	0
29–35 days	0	0
36–42 days	2	1

Regarding the severity of ADRs, there were five mild reports in the placebo group, and 44 mild and eight moderate reports in the PA21 groups, with no severe events being noted. The moderate AEs broke down into five reports of diarrhea (one report in the PA21 1500-mg group, and two reports each in the 2250- and 3000-mg groups), one of abdominal distension (in the 3000-mg group), one of epigastric discomfort (in the 3000-mg group), and one of reduced visual acuity (in the 750-mg group).

During the treatment period, there were a total of eight serious AEs in seven patients in the placebo and PA21 groups. However, all these events were assessed as not related to the investigational product. No deaths were noted in the treatment period.

In ferritin, transferrin saturation, and hemoglobin levels, slight increases were seen in the PA21 groups (Table 5).

**Table 5:** Mean change in laboratory parameters (ferritin levels, serum transferrin saturation, and hemoglobin levels)

	PA21 750-mg group	PA21 1500-mg group	PA21 2250-mg group	PA21 3000-mg group	Placebo group
Ferritin levels, ng/mL, median (quartiles 25 and 75 %) (*N*)					
Baseline	39.9 (18.0, 103.9) (39)	85.0 (32.5, 133.1) (36)	43.1 (18.3, 92.0) (35)	56.2 (25.2, 115.5) (36)	96.0 (30.4, 177.8) (37)
End of treatment	54.4 (33.4, 125.8) (39)	101.5 (46.8, 154.2) (35)	59.1 (37.6, 88.9) (34)	70.7 (38.7, 128.1) (35)	49.1 (26.1, 115.1) (37)
Change from baseline	12.1 (−1.2, 23.9) (39)	18.4 (−8.8, 40.0) (35)	15.2 (6.2, 24.8) (34)	11.4 (−1.8, 34.0) (35)	−14.3 (−52.8, −2.1) (37)
Serum transferrin saturation, %, mean ± SD (*N*)					
Baseline	21.15 ± 8.40 (39)	23.47 ± 10.03 (36)	22.60 ± 10.49 (35)	22.61 ± 10.44 (36)	20.78 ± 7.75 (37)
End of treatment	28.77 ± 12.39 (39)	25.40 ± 8.54 (35)	28.21 ± 8.40 (34)	28.09 ± 11.61 (35)	21.38 ± 7.83 (37)
Change from baseline	7.62 ± 12.79 (39)	2.54 ± 14.31 (35)	5.35 ± 12.01 (34)	5.57 ± 12.56 (35)	0.59 ± 8.88 (37)
Hemoglobin, g/dL, mean ± SD (*N*)					
Baseline	10.55 ± 0.86 (39)	10.54 ± 1.11 (36)	10.84 ± 1.15 (35)	10.64 ± 1.05 (36)	10.46 ± 1.09 (37)
End of treatment	11.08 ± 0.95 (39)	11.05 ± 1.28 (35)	11.37 ± 1.33 (34)	11.20 ± 1.45 (35)	10.39 ± 1.35 (37)
Change from baseline	0.53 ± 0.82 (39)	0.56 ± 1.04 (35)	0.52 ± 1.03 (34)	0.57 ± 0.90 (35)	−0.07 ± 0.77 (37)

In physical examination and 12-lead ECG, no clinically significant changes and no abnormal findings were observed, respectively, in any treatment group.

## Discussion

This is the first randomized, placebo-controlled, double-blind comparative study of PA21 in Japanese hemodialysis patients.

In the present study, the change (mean change adjusted for baseline serum phosphorus level) in serum phosphorus levels from baseline to the end of treatment in all the PA21 groups was significantly different from that in the placebo group. Furthermore, PA21 in the dose range of 750–3000 mg/day decreased serum phosphorus levels in a clear dose-responsive manner.

With PA21 given at 750 mg/day, the mean serum phosphorus level decreased to ≤6 mg/dL from Week 1 of treatment, which is the upper limit of the management target range recommended by the JSDT guidelines [6]. Other phosphate binders administered in minimal doses could not lower serum phosphorus to levels lower than 6 mg/dL from Week 1 [19–21]. With PA21 given at a dose of 1500 mg/day and more, the mean serum phosphorus level decreased to ≤5.5 mg/dL from Week 1 of treatment, which is the upper limit of the management target range recommended by the Kidney Disease Outcomes Quality Initiative guidelines [1].

The efficacy of PA21 was previously confirmed in a Phase II study conducted at multiple sites across Europe and the United States, in which PA21 at dosages of 1000–2500 mg/day was found to be effective in reducing serum phosphorus levels in patients with hyperphosphatemia undergoing hemodialysis [22]. Although both the current study and the previous study showed a dose-responsive serum phosphorus-lowering effect, the efficacy of PA21 was evaluated by comparison with the placebo in the current study, but by comparison with the baseline serum phosphorus concentration in the previous study. Furthermore, a significant serum phosphorus-lowering effect was seen with PA21 at a dose of 750 mg/day or higher in the current study. Efficacy was confirmed at a lower dose in the current study than in the previous study, in which the lowest effective dose was 5.0 g/day, which is equal to 1000 mg/day with iron. In all of the PA21 groups, a significant difference from the placebo group was observed in the cumulative achievement rate of the serum phosphorus level (≤6 mg/dL) at the end of treatment.

These results show that PA21 at a dose of one 250-mg tablet three times/day decreases serum phosphorus levels from the first week of treatment. Furthermore, PA21 demonstrated a dose-dependent phosphorus lowering effect up to 3000 mg/day. The number of patients who discontinued the treatment because their serum phosphorus concentration decreased to below the defined criterion for discontinuation was especially high in the high-dose (2250 and 3000 mg/day) groups. This indicates that PA21 is very effective at lowering serum phosphorus levels.

A lower pill burden is associated with a higher adherence, and furthermore, with a good control of phosphorus levels [23]. Hence, PA21, which shows a serum phosphorus-lowering effect with a single 250-mg tablet/dose, is expected to reduce pill burden and may provide a clinically relevant benefit for serum phosphorus management in dialysis patients.

In both the placebo and PA21 groups, corrected serum calcium levels decreased from Week-3 to baseline, and thereafter, returned to the levels observed at Week-3 in the PA21 groups. Conversely, serum intact-PTH levels increased from Week-3 to baseline, and decreased, thereafter, in the PA21 groups.

PA21 was suggested to secondarily control serum intact-PTH levels by decreasing serum phosphorus levels, without affecting corrected serum calcium levels. The similar intact-PTH changes, which are considered a secondary change related to serum phosphorus levels, were shown for other non-calcium phosphate binders [19–21].

ADRs that occurred at an incidence of ≥5 % in any of the PA 21 groups were diarrhea and constipation, both of which were classified as gastrointestinal disorders. Most cases of diarrhea were mild, and more than half of these cases occurred within 2 days after the initial dose. The severity of the ADRs was mild to moderate, and there were no severe events. The incidence of treatment discontinuation was higher in the high-dose groups. This may be because no dose reduction was possible even in the event of diarrhea, and because higher doses (2250 and 3000 mg/day) were administered from the study start. Although diarrhea occurred frequently in high-dose groups, the incidence of diarrhea was similar between the low-dose (750 and 1500 mg/day) and placebo groups. We consider that the number of cases of excessive phosphorus decrease can be reduced or the development of diarrhea can be avoided by starting treatment at a minimum dose of 750 mg/day.

In laboratory tests, serum ferritin, transferrin saturation, and hemoglobin values tended to increase slightly in the PA21 groups and without a dose-dependent trend. In a clinical pharmacological study evaluating the absorption of iron from PA21 given at a dose of 2000 mg/day, iron absorption after administration of PA21 was very low [24]. Therefore, it is unlikely that iron-related parameters would increase excessively after administration of PA21.

The present study has some limitations. This study only included Japanese patients; thus, the generalizability of the results is limited. However, although the findings of the present study are applicable only to the Japanese population, previous randomized controlled trials have shown a similar serum phosphorus-decreasing effect and safety profile in other ethnic populations [17, 22]. Another limitation is that the placebo did not contain iron; therefore, the participants or the investigators might have discriminated the study drug from the placebo by the stool color. Other limiting factors were the relatively short study duration and the use of placebo as a comparator. Further long-term studies comparing PA21 with other standard therapy in Japanese patients are warranted.

## Conclusion

This study showed that, in the Japanese population, PA21 is an effective and safe phosphate binder that decreases serum phosphorus levels starting at 1 week of treatment when administered at a dose of one 250-mg tablet three times/day. Furthermore, PA21 demonstrated a dose-dependent phosphorus lowering effect up to 3000 mg/day. Therefore, PA21 is useful as a new treatment alternative with a relatively low pill burden for Japanese hemodialysis patients with hyperphosphatemia.
